# The Relationship between Attachment Styles and Compulsive Online Shopping: The Mediating Roles of Family Functioning Patterns

**DOI:** 10.3390/ijerph19138162

**Published:** 2022-07-03

**Authors:** Eleonora Topino, Marco Cacioppo, Alessio Gori

**Affiliations:** 1 Department of Human Sciences, LUMSA University of Rome, Via della Traspontina 21, 00193 Rome, Italy; m.cacioppo@lumsa.it; 2 Department of Health Sciences, University of Florence, Via di San Salvi 12, Pad. 26, 50135 Florence, Italy; alessio.gori@unifi.it

**Keywords:** shopping addiction, technological addiction, behavioral addiction, problematic online buying, family, attachment, parallel mediation

## Abstract

The rapid expansion of e-commerce has made the buying experience faster, potentially anonymous, and without limits of space and time. While this may produce benefits, for some individuals, online shopping can become an addiction. Therefore, the present study aimed to explore the psychological factors that may be associated with Compulsive Online Shopping, with a specific focus on the role of Attachment Styles and Family Functioning patterns as risk or protective factors. The study involved a sample of 306 participants (*M_age_* = 31.86 years, *SD* = 11.925) who filled out an online survey consisting of the Compulsive Online Shopping Scale, Relationship Questionnaire, Family Adaptability and Cohesion Evaluation Scales-IV, as well as a demographic questionnaire. The results showed two significant parallel mediation models. In the first one, Secure Attachment was negatively and significantly related to Compulsive Online Shopping, with the mediation of Cohesion and Enmeshed Family Functioning. In the second one, Fearful Attachment was positively and significantly related to Compulsive Online Shopping, with the mediation of Cohesion and Enmeshed Family Functioning. Important implications for preventive activity and tailored interventions may emerge from these data.

## 1. Introduction

The diffusion of the Internet and the rapid expansion of e-commerce activities have revolutionized the shopping experience of buyers, who are exposed to personalized and immersive activities linked to a wide range of products, potentially anonymously, without limits of space and time (see Rose and Dhandayudham [1] for a review). Furthermore, as the possibility of shopping online has increased, disorders related to compulsive online shopping have also emerged [2]. Compulsive Online Shopping, also called Online Shopping Addiction, is a technological addiction [1,3] characterized by excessive and compulsive buying behaviors implemented through the Internet that the person cannot control and continues to perpetuate despite the negative consequences resulting from it on an economic, social, emotional level and in several important areas of the individual’s life [4,5]. The phenomenon causes clinically significant distress and leads to impairments in various areas of functioning [6,7]; it has, in fact, been associated with legal and fiscal difficulties, alterations in the sleep–wake cycle, somatic symptoms [8], lower life satisfaction [9], and a general decline in quality of life [10]. Although scientific research has focused on the investigation of Compulsive Shopping in loco (see Black [11] for a review), the exploration of the psychological antecedents for its online declination is still scarce. In fact, Compulsive Online Shopping is a distinct phenomenon [12] that also incorporates the characteristics of cyberspace where it occurs (speed, anonymity, convenience, etc.) [1], where the object of dependence is more and easily accessible, a wider offer is proposed, and social control may be reduced: the online market, therefore, potentially offers greater ease in developing susceptibility to compulsive buying for people at risk compared to on-site retail sales [13]. Therefore, given the growing importance of the Internet in consumer behavior, the study of the variables that influence the tendency to compulsive Internet shopping acquires fundamental importance in favouring effective health risk and impact management [14]. The general aim of the present research was to investigate the psychological factors that may be associated with Compulsive Online Shopping by specifically focusing on the roles of attachment styles and the perception of family functioning in contributing to the phenomenon.

Attachment refers to “*the lasting psychological connectedness between human beings*” (p. 194) [15]. Although attachment theory originally focused on relationships with caregivers, its developments have allowed for the elaboration of a theoretical perspective that allows explaining relational cognitions and behaviors throughout the life span [16,17]. The quality of attachment in the adult is defined by the balance between being able to seek help in times of difficulty and leveraging own’s resources to overcome challenging situations [17], and plays a protective or vulnerable role in the well-being of the individual [16]. Previous authors have elaborated the hypothesis that addictions can be read as an attachment disorder [18,19]. Indeed, insecure attachment plays a key role in both susceptibility [20] and severity [21] of the addiction, and has shown significant associations not only with problematic shopping [22], but also with other behavioral addictions [23], including those related to the Internet and the use of technology [24,25,26,27,28], as well as with alcohol and substance abuse [29,30]. On the other hand, secure attachment represents a protective factor both for addictions (e.g., [26]), and for psychopathological disorders in general (e.g., [31,32]). Furthermore, attachment styles in adults can influence the quality of family relationships [33], another element that has been shown to have weight in relation to the onset of dysfunctional behaviors [34,35]. 

Family functioning refers to the quality of emotional connection, rules, and communication in the family context [36]. A solid body of empirical research documents that good family functioning is a protective factor against psychopathology [37] and positively correlates with mental health levels [38]. Conversely, poor family functioning is associated with major physical and psychological problems [39], such as asthma [40], diabetes [41], dementia [42], mood disorders, and anxiety disorders [43,44], to name a few. In the context of addictions, dysfunctional family functioning is frequently found in subjects with alcohol and substance abuse [45,46] and with behavioural addictions, such as gambling disorder [47], problematic smartphone use [48], Internet addiction [49], as well as compulsive shopping [50]. Furthermore, recent evidence showed a negative correlation between the family functioning of individuals with drug addiction and their relapse tendency [51], supporting the importance of family collaboration during the treatment process [52].

On this basis, the aim of this research was to contribute to the knowledge of the effect of Attachment Styles and Family Functioning in contributing to Compulsive Online Shopping. Therefore, a series of parallel mediation models were implemented to analyse the relationships between Secure, Dismissing, Preoccupied, or Fearful Attachment and Compulsive Online Shopping, exploring the mediation role of Cohesion, Flexibility, Disengaged, Enmeshed, Rigid, and Chaotic family patterns in these pathways. More specifically, it was hypothesised that Secure Attachment was negatively associated with Compulsive Online Shopping, contrary to insecure ones, for which a positive relationship is assumed. The mediation role of Family Functioning in these pathways was also explored.

## 2. Materials and Methods

### 2.1. Participants, Procedure, and Ethics

The study involved a sample of 306 participants who responded to an online survey. Their age ranged from 18 to 72 years (*Mage* = 31.86 years, *SD* = 11.925), and most of them were female (76%), single (66%), student (39%), with a university degree (45%), as shown in Table 1. The recruitment process was implemented through an online, snowball sampling design by advertising the study on the authors’ various social networks with a recruitment message that included an anonymous link that took participants to the survey hosted on Google Forms. The participants were informed about the general aim of the study and had given their informed consent (electronically) before starting the survey. Confidentiality and anonymity were guaranteed, and they could withdraw from the study at any time. All of the procedures performed in the study have been approved by the Ethics Committee for Scientific Research (CERS) of the LUMSA University of Rome, Rome, Italy.

### 2.2. Measures

#### 2.2.1. Compulsive Online Shopping Scale (COSS)

The Compulsive Online Shopping Scale (COSS; Manchiraju, Sadachar, and Ridgway [4]; Italian version: Gori, Topino, and Casale [12]) is a self-report scale used to assess compulsive shopping behaviours on the Internet. It consists of 28 items, rated on a seven-point Likert scale (from 1 = “Strongly Disagree” to 7 = “Strongly Agree”) and grouped into seven factors: Salience (Cronbach’s α in the present sample of 0.87), Mood modification (Cronbach’s α in the present sample of 0.91), Conflict (Cronbach’s α in the present sample of 0.84), Tolerance (Cronbach’s α in the present sample of 0.85), Relapse (Cronbach’s α in the present sample of 0.88), Withdrawal (Cronbach’s α in the present sample of 0.84), and Problems (Cronbach’s α in the present sample of 0.82). In this study, the total score of the Italian version was used, which showed excellent internal consistency in the present sample (the Cronbach’s α value is 0.95).

#### 2.2.2. Relationship Questionnaire (RQ)

The Relationship Questionnaire (RQ; Bartholomew and Horowitz [17]; Italian version: Carli et al. [53]) is a self-report instrument for the evaluation of attachment patterns. It consists of 28 items, rated on a seven-point Likert scale (from 1 = “It does not describe me at all” to 7 = “It very much describes me”) and allows for the assessment of four styles: Secure, Dismissing, Preoccupied, and Fearful. Since the four attachment styles are assessed with a single item, the alpha coefficient cannot be calculated. However, the RQ is a valid and well-validated measure of attachment among adults showing good test-retest reliability [54], as well as strong psychometric properties in different cultures [55] and among the general population [17] and clinical samples [56], and the Italian version administered in this study is effectively and frequently used in the Italian context (e.g., [49,57]).

#### 2.2.3. Family Adaptability and Cohesion Evaluation Scales-IV (FACES IV)

The Family Adaptability and Cohesion Evaluation Scales-IV (FACES IV; Olson [58]; Italian version: Baiocco et al. [59]) is a self-report scale used to assess family functioning based on the Circumplex Model of Marital and Family Systems [58]. It consists of 42 items five-point Likert scale (from 1 = “Strongly Disagree” to 5 “Strongly Agree”), grouped into six subscales, two of which indicate features of the balanced functioning (cohesion and flexibility), while four concern the unbalanced one (Enmeshed, Disengaged, Chaotic, and Rigid). In the present study the Italian version was used, for which the six scales showed satisfactory internal consistency in the present sample (Cohesion, α = 0.90; Flexibility, α = 0.78; Enmeshed, α = 0.76; Disengaged, α = 0.75; Chaotic, α = 0.66; Rigid α  = 0.0.70).

### 2.3. Data Analysis

The Statistical Package for the Social Sciences (SPSS) version 21.0 (IBM, Armonk, NY, USA) for Windows was used to analyse the collected data. A *p* < 0.05 value was set as the threshold of statistical significance in the present study. Pearson correlation analysis was implemented to evaluate the associations between the variables by interpreting the effect as small if the coefficient value lies between ±0.01 and ±0.29, medium if the coefficient value lies between ±0.30 and ± 0.49, and large if the coefficient value lies between ±0.50 and ±1.00 [60]. To assess the effect of the different Attachment Styles on Compulsive Online shopping, also by exploring the role of the Family Functioning patterns in this relationship, several parallel mediation models were tested using the macro-program PROCESS 3.4 [61]. Subsequently, follow-up models including only the significant relationships were performed to more accurately define the obtained results. For each regression coefficient included in the models, the 95% confidence interval (CI) was calculated. Finally, the statistical stability of the models was probed by performing the Bootstrapping procedures with a 95% Confidence Interval (CI) at 5000 samples, which supported the significance of the effect when the CI (from lower limit confidence interval [Boot LLCI] to upper limit confidence interval [Boot ULCI]) does not include zero.

## 3. Results

Significant correlations emerged among some variables included in this study, as showed in Table 2. Specifically, Compulsive Online Shopping was significantly and negatively associated with Secure Attachment (*r* = −0.172, *p* < 0.01), Cohesion (*r* = −0.199, *p* < 0.01), and Flexibility (*r* = −0.134, *p* < 0.05). On the other hand, Compulsive Online Shopping showed significant and positive relationship with Fearful Attachment (*r* = 0.155, *p* < 0.05), and Disengaged (*r* = 0.216, *p* < 0.01), Enmeshed (*r* = 0.295, *p* < 0.01), Rigid (*r* = 0.235, *p* < 0.01), and Chaotic (*r* = 0.215, *p* < 0.01) Family Functioning patterns.

A series of parallel mediations was performed to investigate the role of the different Attachment Styles and Family Functioning patterns in contributing to Compulsive Online Shopping (see Table 3). The results showed that the effect of Preoccupied Attachment on Compulsive Online Shopping was nonsignificant (*β* = 0.09, *p* = 0.102; LLCI = −0.008—ULCI = 0.092), as it was for Dismissing Attachment (*β* = 0.052, *p* = 0.365; LLCI = −0.028—ULCI = 0.075).

**Figure 1:**
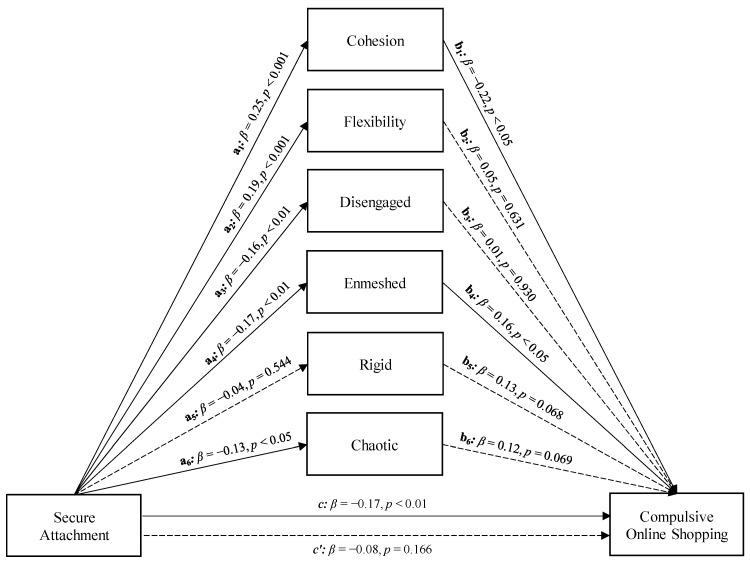
Model 1: a parallel mediation model concerning the relationship between Secure Attachment and Compulsive Online Shopping, with different features of family functioning as parallel mediators.

On the other hand, Secure Attachment showed a significant total effect on Compulsive Online Shopping (Path *c* in Figure 1; *β* = −0.17, *p* < 0.01, LLCI = −0.117—ULCI = −0.025). Furthermore, Secure Attachment was significantly associated with Cohesion (Path *a*_1_ in Figure 1; *β* = 0.16, *p* < 0.001, LLCI = 0.515—ULCI = 1.269), Flexibility (Path *a*_2_ in Figure 1; *β* = 0.19, *p* < 0.001, LLCI = 0.232—ULCI = 0.858), Disengaged (Path *a*_3_ in Figure 1; *β* = −0.16, *p* < 0.01, LLCI = −0.697—ULCI = −0.119), Enmeshed (Path *a*_4_ in Figure 1; *β* = −0.17, *p* < 0.01, LLCI = −0.695—ULCI = −0.151), and Chaotic (Path *a*_6_ in Figure 1; *β* = −0.13, *p* < 0.05, LLCI = −0.592—ULCI = −0.048) Family Functioning patterns. Only Cohesion (Path *b*_1_ in Figure 1; *β* = −0.22, *p* < 0.05, LLCI = −0.052—ULCI = −0.001) and Enmeshed Family Functioning (Path *b*_4_ in Figure 1; *β* = 0.16, *p* < 0.05, LLCI = 0.003—ULCI = 0.051) were, in turn, significantly associated with Compulsive Online Shopping. Entering the Family Functioning patterns in the model parallelly, only Cohesion and Enmeshed Family Functioning played a statistically significant role in the relationship between Secure Attachment and Compulsive Online Shopping, at a level whose direct effect was nonsignificant after controlling the mediators (path *c’* in Figure 1; *β* = −0.08, *p* = 0.166; LLCI = −0.078—ULCI = 0.014). Therefore, a total mediation occurred (*R*^2^ = 0.152, *F*(7, 298) = 7.625, *p* < 0.001; see Figure 1). Furthermore, the significance of the model was supported by the bootstrap procedure, which confirmed the statistical relevance of the indirect effect (Boot LLCI = −0.065—Boot ULCI = −0.017).

**Figure 2:**
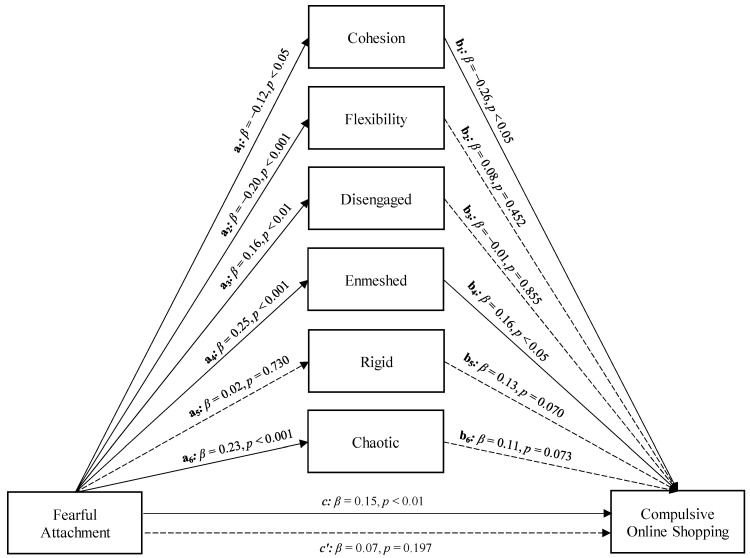
Model 2: a parallel mediation model concerning the relationship between Fearful Attachment and Compulsive Online Shopping, with different features of family functioning as parallel mediators.

Parallelly, Fearful Attachment showed a significant total effect on Compulsive Online Shopping (Path *c* in Figure 2; *β* = 0.15, *p* < 0.01, LLCI = 0.022—ULCI = 0.134). Furthermore, Fearful Attachment was significantly associated with Cohesion (Path *a*_1_ in Figure 2; *β* = −0.12, *p* < 0.05, LLCI = −0.967—ULCI = −0.023), Flexibility (Path *a*_2_ in Figure 2; *β* = −0.20, *p* < 0.001, LLCI = −1.062—ULCI = −0.299), Disengaged (Path *a*_3_ in Figure 2; *β* = 0.16, *p* < 0.01, LLCI = 0.157—ULCI = 0.862), Enmeshed (Path *a*_4_ in Figure 2; *β* = 0.25, *p* < 0.001, LLCI = 0.420—ULCI = 1.072), and Chaotic (Path *a*_6_ in Figure 2; *β* = 0.23, *p* < 0.001, LLCI = 0.347—ULCI = 0.998) Family Functioning patterns. Only Cohesion (Path *b*_1_ in Figure 2; *β* = −0.26, *p* < 0.05, LLCI = −0.056—ULCI = −0.006) and Enmeshed (Path *b*_4_ in Figure 2; *β* = 0.16, *p* < 0.05, LLCI = 0.003—ULCI = 0.051) Family Functioning were, in turn, significantly associated with Compulsive Online Shopping. Entering the Family Functioning patterns in the model parallelly, only Cohesion and Enmeshed Family Functioning played a statistically significant role in the relationship between Fearful Attachment and Compulsive Online Shopping, at a level whose direct effect was nonsignificant after controlling the mediators (path *c’* in Figure 2; *β* = 0.07, *p* = 0.197; LLCI = −0.019—ULCI = 0.094). Therefore, a total mediation occurred (*R*^2^ = 0.151, *F*(7, 298) = 7.581, *p <* 0.001; see Figure 2). Furthermore, the significance of the model was supported by the bootstrap procedure, which confirmed the statistical relevance of the indirect effect (Boot LLCI = 0.014—Boot ULCI = 0.074).

The follow-up models confirmed the results obtained from the previous analyses. Indeed, by inserting only Cohesion and Enmeshed Family Functioning as parallel mediators in the relationship between Secure attachment and Compulsive online shopping, the association between secure attachment and both the mediators was confirmed (*β* = 0.25, *p* < 0.001, LLCI = 0.515—ULCI = 1.269 and *β* = −0.17, *p* < 0.01, LLCI = −0.695—ULCI = −0.151, respectively) as well as that of the latter with Compulsive online shopping (*β* = 0.23, *p* < 0.001, LLCI = 0.347—ULCI = 0.998 and *β* = 0.23, *p* < 0.001, LLCI = 0.347—ULCI = 0.998, respectively). Entering both Cohesion and Enmeshed Family Functioning as mediators simultaneously, the direct effect between Secure attachment and Compulsive online shopping became nonsignificant (*β* = −0.08, *p* = 0.139; LLCI = −0.081—ULCI = 0.011), suggesting for a total mediation (*R*^2^ = 0.126, *F*(3, 302) = 14.568, *p < 0*.001). The bootstrap procedure confirmed the statistical relevance of the indirect effect (Boot LLCI = −0.143—Boot ULCI = −0.043).

Furthermore, by inserting only Cohesion and Enmeshed Family Functioning as parallel mediators in the relationship between Fearful attachment and Compulsive online shopping, the association between fearful attachment and both the mediators was confirmed (*β* = −0.12, *p* < 0.05, LLCI= −0.967–ULCI= −0.023 and *β* = 0.25, *p* < 0.01, LLCI = 0.420—ULCI = 1.072, respectively) as well as that of the latter with Compulsive online shopping (*β* = −0.17, *p* < 0.01, LLCI = −0.028—ULCI = −0.008 and *β* = 0.27, *p* < 0.001, LLCI = 0.027—ULCI = 0.064, respectively). Entering both Cohesion and Enmeshed Family Functioning as mediators simultaneously, the direct effect between Fearful attachment and Compulsive online shopping became nonsignificant (*β* = 0.07, *p* = 0.228; LLCI = −0.021—ULCI = 0.090), suggesting for a total mediation (*R*^2^ = 0.124, *F*(3, 302) = 14.284, *p < 0*.001). The bootstrap procedure confirmed the statistical relevance of the indirect effect (Boot LLCI = 0.042—Boot ULCI = 0.138).

## 4. Discussion

Due to the growing digitalisation and use of the Internet, compulsive shopping has also been translated into the digital world, where, however, the purchasing behaviour occurs much more quickly, simultaneously, and in the absence of time and place restrictions (see Niedermoser et al. [62] for a review). The aim of this study was the analysis of the psychological variables that may contribute to Compulsive Online Shopping, with a specific focus on the exploration of the role of Attachment Styles and the perception of Family Functioning as risk or protective factors.

The results showed a significant and negative association between secure attachment and Compulsive Online Shopping, in line with what was hypothesised. This further supports the key role of attachment security in protecting mental health, enriching a framework of previous research highlighting important relationships with resilience, emotion regulation, and well-being [63,64]. Furthermore, the data have shown that Secure Attachment also had an indirect effect on Compulsive Online Shopping through the significant parallel mediation of Cohesion and Enmeshed Family Functioning. Such findings, therefore, confirm that attachment theory provides a solid and empirically grounded framework for reading and understanding important aspects of interpersonal functioning [65]. Specifically, Secure Attachment had a positive effect on Cohesion which in turn was negatively associated with Compulsive Online Shopping, in the opposite way to what was shown with Enmeshed Family Functioning. On the one hand, cohesion refers to the “*emotional bonding among family members and the feeling of closeness that is expressed by feelings of belonging and acceptance within the family*” (p. 306) [66] and may play a significant role in preventing the development of addictions [67,68], including those that occur online (e.g., [69]), consistently with our data. On the other hand, Enmeshed Family Functioning is characterised by excessive closeness between family members, with poorly defined roles and boundaries [70], and represents an unbalanced pattern that has been associated with mental illness [71].

Concerning insecure attachment styles, the data partially support the hypothesis: indeed, only the Fearful pattern showed a significant and positive relationship with Compulsive Online Shopping. Individuals with Fearful attachment have negative internal working models both with respect to the self and the other; they consider themselves unlovable and believe that others will not be able to satisfy their needs [17]. Previous evidence has shown an association between the fearful pattern and negative psychological health outcomes, such as social anxiety, loneliness, problematic smartphone use, depression, and rumination [72,73,74]. In this framework, therefore, subjects with Fearful Attachment might use Compulsive Online Shopping as an external maladaptive coping strategy to cope with negative internal experiences, in line with the self-medication hypothesis of addictive disorders [75,76,77]. Consistently, several studies conceptualised compulsive online shopping as a result of escape and compensation mechanisms for dysregulated negative states, such as anxiety and stress [13,78,79,80]. Data have shown a significant parallel mediation of Cohesion and Enmeshed Family Functioning, such that Fearful Attachment showed a positive effect on Enmeshed Family Functioning, which in turn was positively associated with Compulsive Online Shopping, as opposed to what happened with Cohesion. This is consistent with recent studies on problematic smartphone use, for which Cohesion and Enmeshed Family Functioning were the best predictors [48]. Therefore, adults with Fearful Attachment tend to perceive the context as poorly structured in terms of roles and boundaries and experience greater difficulties in creating emotional bonds, and this could lead them to seek a form of escape and compensation from unsatisfactory relationships in Compulsive Online Shopping [81].

The present study has some limitations that should be highlighted. First, the cross-sectional design implies the need to be cautious in interpreting the causal relationships between the variables. The implementation of longitudinal studies could be an important challenge for future research. Moreover, the gender imbalance in the sample could be a further limitation of the research. Future research could recruit more balanced samples to confirm the generalisability of these data for both men and women. In addition, only self-report measures were used to collect the data, and this may expose to well-known bias (e.g., social desirability). The integration of these instruments with other methods (e.g., interviews) could allow us to overcome this limitation in future research. Furthermore, the present research focused on the analysis of the variables which may be related to Compulsive Online Shopping (a form of behavioural addiction), while the associations between Attachment styles and Family Functioning patterns with different types of shoppers are not necessarily problematic (e.g., Surgical shopper, Power shopper, etc.) [82] has not been explored. Investigating the relationships between Compulsive Online Shopping Attachment styles and Family Functioning patterns may be an important challenge for future research. Furthermore, the criterion of significance was set at *p* < 0.05 in this study, and no protocols have been developed to verify the risk of type I error. Therefore, although the preliminary results obtained appear consistent with other data on the topic of addictions, these findings will have to be confirmed in future research by more in-depth analyses (e.g., by performing the Bonferroni correction or structural equation modelling).

Finally, the absence of a clinical sample and a snowball sampling design may be considered factors that may limit the generalization of these results. In fact, even if convenience sampling can be a quick, flexible, and useful way to elaborate preliminary results [83], future research must confirm such findings in more representative samples, both from the general population and the clinical one. Despite these limitations, this study presents useful preliminary data that may provide important input for future research, which could replicate and expand these results on more representative samples, and may suggest significant data, which can be kept in mind for the elaboration of therapeutic and preventive activities focused on Compulsive Online Shopping.

## 5. Conclusions

The scientific interest in behavioural addictions is constantly evolving, with a focus not only on offline and onsite addictions such as gambling disorder [20,84] or exercise addiction [85,86,87] but also on those related to the digital world (e.g., [48,88]). Indeed, although the Internet offers numerous advantages by facilitating communications, speeding up the circulation of information, and expanding access to services, it can also become the site of some problematic and dysfunctional behaviours. Therefore, the analysis of the antecedents of technological addictions is of great scientific interest and clinical relevance. This study focused on Compulsive Online shopping, highlighting the protective role of Secure Attachment and Family Functioning characterized by Cohesion, but also how Fearful Attachment and Enmeshed Family Functioning can represent risk factors. Such data fall within a framework that supports the role of the familiar context and attachment for the treatment of addictions [89,90] and can provide information for favouring effective health risk management and elaborating tailored interventions and specific prevention activities for Compulsive Online Shopping to support both individual and public health.

## Figures and Tables

**Table 1:** Demographic characteristics of the sample (*n =* 306).

Characteristics		*M* ± *SD*	*n*	%
*Age* (years)		31.86 ± 11.925		
*Gender*				
	Females		234	76
	Males		72	24
*Marital Status*				
	Single		202	66
	Married		55	18
	Cohabiting		36	12
	Separated		2	1
	Divorced		10	3
	Widowed		1	0
*Education*				
	Middle School diploma		9	3
	High School diploma		92	30
	University degree		139	45
	Master’s degree		50	16
	Post-lauream specialization		16	5
*Occupation*				
	Student		120	39
	Working student		37	12
	Employee		88	29
	Freelance		21	7
	Manager		2	1
	Entrepreneur		5	2
	Trader		6	2
	Artisan		3	1
	Unemployed		17	6
	Retired		7	2

**Table 2:** Correlation Matrix.

	1	2	3	4	5	6	7	8	9	10	11
1. COSS	1										
2. RQ (1)	**−0.172** **	1									
3. RQ (2)	0.094	−0.292 **	1								
4. RQ (3)	0.155 **	−0.169 **	0.258 **	1							
5. RQ (4)	0.052	−0.236 **	0.088	0.01	1						
6. FACES-IV (1)	−0.199 **	0.258 **	−0.034	−0.118 *	0.042	1					
7. FACES-IV (2)	−0.134 *	0.193 **	−0.104	−0.197 **	0.115 *	0.817 **	1				
8. FACES-IV (3)	0.216 **	−0.157 **	0.196 **	0.161 **	0.178 **	−0.390 **	−0.224 **	1			
9. FACES-IV (4)	0.295 **	−0.173 **	0.139 *	0.250 **	0.006	−0.062	−0.106	0.289 **	1		
10. FACES-IV (5)	0.235 **	−0.035	0.110	0.020	0.025	0.021	0.207 **	0.342 **	0.485 **	1	
11. FACES-IV (6)	0.215 **	−0.132 *	0.276 **	0.227 **	0.076	0.033	−0.010	0.384 **	0.448 **	0.188 **	1

Note: Bold values indicate significant *p*-values. ** Correlation is significant at the 0.01 level (2-tailed). * Correlation is significant at the 0.05 level (2-tailed). COSS = Compulsive Online Shopping Scale; RQ (1) = Secure attachment (Relationship Questionnaire); RQ (2) = Fearful (Relationship Questionnaire); RQ (3) = Preoccupied (Relationship Questionnaire); RQ (4) = Dismissing (Relationship Questionnaire); FACES-IV (1) = Cohesion (Family Adaptability and Cohesion Evaluation Scales-IV); FACES-IV (2) = Flexibility (Family Adaptability and Cohesion Evaluation Scales-IV); FACES-IV (3) = Disengaged (Family Adaptability and Cohesion Evaluation Scales-IV); FACES-IV (4) = Enmeshed (Family Adaptability and Cohesion Evaluation Scales-IV); FACES-IV (5) = Rigid (Family Adaptability and Cohesion Evaluation Scales-IV); FACES-IV (6) = Chaotic (Family Adaptability and Cohesion Evaluation Scales-IV).

**Table 3:** Models effect indices.

Independent Variable	Parallel Mediators	Dependent Variable	Total Effect	Direct Effect	Indirect Effect [Bootstrapping 95% CI]
Secure Attachment	Family Functionings	Compulsive Online Shopping	−0.071	−0.032	**−0.039** [−0.0649; −0.0172]
Preoccupied Attachment	Family Functionings	Compulsive Online Shopping	0.042	0.010	−0.031 [−0.0325; 0.0538]
Fearful Attachment	Family Functionings	Compulsive Online Shopping	0.078	0.037	**0.041** [0.0138; 0.0742]
Dismissing Attachment	Family Functionings	Compulsive Online Shopping	0.024	0.061	**0.002** [−0.0203; 0.0277]

Note: Bold indicate significant values.

## Data Availability

The data presented in this study are available on request from the corresponding author. The data are not publicly available, for reasons of privacy.
